# Acute fatty liver of pregnancy: a case report

**DOI:** 10.1186/s12884-019-2405-5

**Published:** 2019-07-22

**Authors:** Enesia Ziki, Shingi Bopoto, M. G. Madziyire, D. Madziwa

**Affiliations:** 1 Department of Obstetrics and Gynaecology, University of Zimbabwe, College of Health Sciences, Harare, Zimbabwe; 2 Department of Histopathology, University of Zimbabwe, College of Health Sciences, Harare, Zimbabwe

**Keywords:** Acute fatty liver of pregnancy

## Abstract

**Background:**

Acute Fatty Liver of Pregnancy (AFLP) is a rare, catastrophic disease affecting women in the third trimester of pregnancy or in the post-partum period. It is usually a diagnosis of exclusion and requires a strong index of suspicion for a timely diagnosis and prompt intervention.

**Case presentation:**

We report a case of AM, an 18 year patient, in her first pregnancy at 35 weeks gestation who presented with nausea, vomiting and jaundice. She had a vaginal delivery following spontaneous preterm labour. A clinical diagnosis of acute fatty liver of pregnancy was made on the 3rd day post-delivery. The post-delivery course was complicated by a deterioration of clinical symptoms with worsening hepatorenal function and development of encephalopathy. The patient died 3 days after admission and the diagnosis was confirmed on post-mortem and histology.

**Conclusion:**

Delay in the diagnosis is associated with morbid complications with high mortality and this case highlights the importance of a high index of suspicion of the condition in women presenting with jaundice in pregnancy.

## Background

AFLP is a disease of the third trimester that is unique to human pregnancy and was described by Sheehan in 1940 [1]. The condition was associated with high mortality rates but this has improved because of early diagnosis and prompt delivery of the foetus [2]. The approximate incidence of AFLP is 1: 7,000 to 1:20,000 [3]. Conditions unique to pregnancy that cause liver dysfunction include intrahepatic cholestasis of pregnancy, pre-eclampsia, Haemolysis Elevated Liver enzymes Low Platelet count (HELLP) syndrome and AFLP. While intrahepatic cholestasis of pregnancy (ICP) and pre-eclampsia are frequently seen, AFLP is rare and potentially life-threatening. The pathogenesis of AFLP remains unclear but there is emerging evidence of the genetic basis of AFLP where defective mitochondrial fatty acid beta-oxidation in the foetus is implicated in some cases of AFLP [3]. We present a case where the diagnosis was delayed with subsequent poor outcome.

## Case presentation

**Fig. 1:**
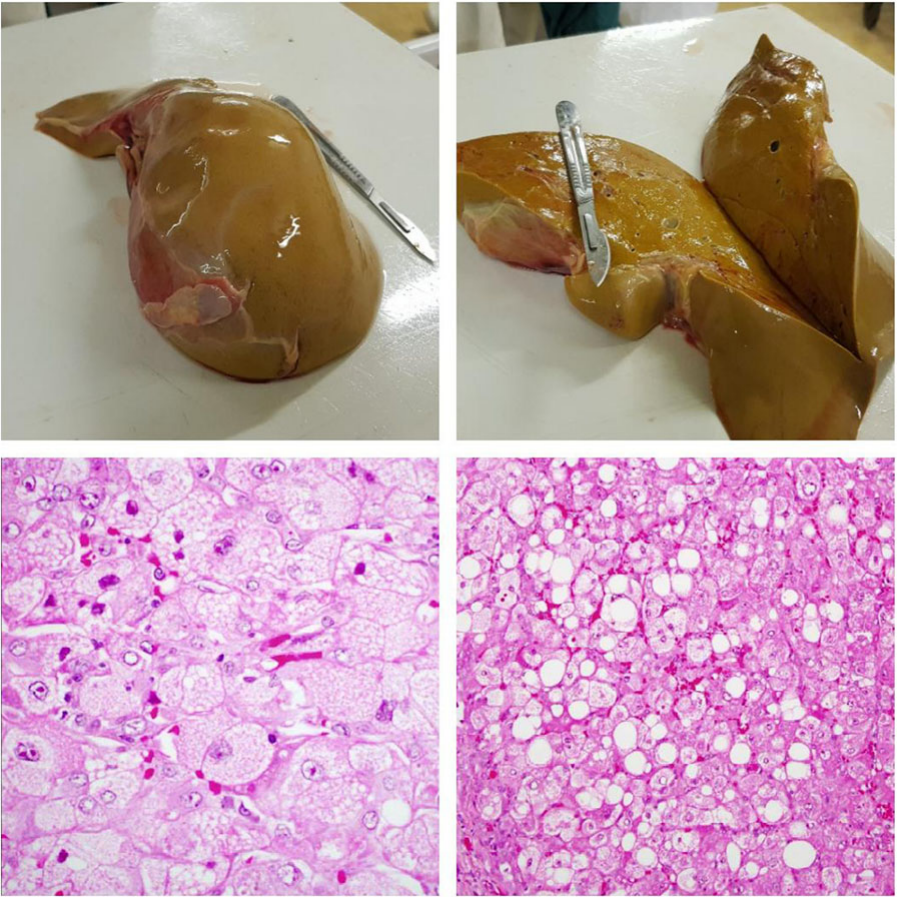
from left to right: Macroscopic appearance of the liver showing pale shrunken liver weighing 1 kg

An 18 year old primiparous with an estimated gestational age (EGA) of 35 weeks and 6 days presented to the hospital as a referral from a local clinic in preterm labour. She had been generally unwell for 4 days prior to presentation and reported that she had nausea, vomiting and yellowing of the eyes for the same duration with no pruritus. There was no history of diarrhoea or flu like symptoms. She was booked and the Antenatal Clinic (ANC) visits were unremarkable. She had no history of travel to a malaria endemic area. Human Immunodeficiency Virus (HIV) status was negative. She had no chronic illnesses and gave no history of paracetamol, asprin, sodium valproate or herbal medicine ingestion. Clinical examination revealed a deeply jaundiced patient who was fully conscious at initial examination. The blood pressure, pulse and temperature were 118/69 mmHg, 101 bpm and 37.1 degrees Celsius respectively. Respiratory and cardiovascular examinations were normal. The abdomen was soft and there was no hepatomegaly or splenomegaly. The height of fundus was 35 weeks and the foetal heart was present and normal. She was draining thin meconium stained liquor at presentation and progressed to a spontaneous vaginal delivery of a baby boy with an Apgar score of 9/10 and birth weight of 2,230 g. She did not have postpartum haemorrhage. The patient was admitted to early labour ward for observation. The vital signs remained normal. Comparison of haematogical and renal tests done on admission and then 48 h later showed a rising white blood cell count (WBC) (26 to 50 cells/mm3), falling haemoglobin (11.1 to 4.7 g/dl) hyponatremia (sodium 134.5 to 127 mmol/l), rising urea (14.3 to 17.9), rising potassium (4.87 to 5.6 mmol/l). Platelets (PLT) remained normal (189-221 × 103). The liver function tests showed elevated alkaline phosphatase (ALP) (331 to 277 IU/L) and gamma glutamyl transpeptidase (GGT) (228 to 206 IU/L) with minimally elevated aspartate aminotransferase (AST) (39 to 52 U/l) and alanine aminotransferase (ALT) (48 TO 39 U/l). Total protein and albumin were normal. Total bilirubin was not available. Hepatitis A and B were negative. Rapid Diagnostic Test (RDT) for malaria was negative. The urine was dark but urinalysis was negative. She had several episodes of hypoglycaemia (1.1–2.3 mmol/l) which were corrected with 50% dextrose followed by infusion of 10% dextrose 8 hourly. Coagulation studies, uric acid and were requested on day 3 but were not available at the hospital. She was transfused 3 units of packed cells. The clinical condition of the patient deteriorated on the third day post-delivery as she became confused and she started vomiting coffee ground material. The patient deceased on the 3rd day post-delivery. The neonate was referred to paediatricians for ongoing care. Post mortem and histology results confirmed the diagnosis (Fig. 1).

Cut surface of the liver with no nodularity seen.

High power microscopic appearance of the liver showing hepatocyte with foamy and granular cytoplasm.

High power microscopic appearance of the liver showing a dual picture of macro and micro vesicular steatosis characterized. Macro and Micro vesicular steatosis is sometimes seen in acute fatty liver of pregnancy. No cholestasis or necrosis is seen and there is minimal inflammation.

## Discussion

A case of a patient with suspected AFLP and poor outcome with the diagnosis confirmed at post-mortem is presented. This case highlights the importance of a high index of suspicion of the condition in women presenting with jaundice in pregnancy. Other differential diagnoses of jaundice occurring during pregnancy include viral hepatitis, preeclampsia, cholelithiasis and intrahepatic cholestasis of pregnancy (ICP) [4]. The clinical presentation and laboratory findings of AFLP are vague and nonspecific, and pose a diagnostic challenge [5]. It is important to always consider life threatening differentials which may require prompt delivery and intensive care. Whilst HELLP syndrome and AFLP usually complicate the third trimester of pregnancy, HELLP syndrome (1 in 5000) is seen more frequently than AFLP (1 in 13000) [4, 6]. In our case, the patient was at risk for both conditions as she was young and nulliparous. The blood pressure was however normal and urinalysis was negative for proteinuria. Key features of jaundice, hepatic encephalopathy, and the episodes of hypoglycaemia, normal ALT, AST, PLT and a very high WBC as well as the coagulopathy shown by upper gastrointestinal bleeding made the diagnosis of AFLP more likely. Other tests that were not available to us were coagulation studies which would have aided in the diagnosis of the coagulopathy and timely supportive care with blood products. Viral hepatitis also presents with jaundice but is characterised by a generally unwell patient with fever, nausea, vomiting and markedly elevated aminotransferases. Patients with intrahepatic cholestasis of pregnancy commonly complain of pruritus and their serum bilirubin levels do not usually exceed 6 mg/dl [2]. Ingestion of drugs and herbal remedies that could lead to hypoglycaemia were ruled out from the history. Patients with cholelithiasis, in addition to the jaundice, also have pain the right upper quadrant as well as fever and an ultrasound scan aids in the diagnosis. Cholelithiasis and viral hepatitis may occur at any time during pregnancy unlike AFLP which is usually diagnosed in the third trimester as noted earlier [7]. Sepsis was unlikely as the patient had no tachycardia or hypotension and remained normothermic. Other differential diagnoses were excluded in our case based on the symptoms, the timing of the presentation and investigations that were available. Our patient had a profound fall in the haemoglobin. It was difficult to attribute this fall due to haemolysis alone as we were unable to get the results for bilirubin levels, reticulocyte count and a peripheral blood smear. Blood loss into the gastrointestinal tract may be difficult to ascertain but may account for the fall.

The definitive management of AFLP is rapid delivery of the foetus and supportive intensive care. As seen in our case, jaundice, liver dysfunction, and coagulopathy may progress for 1 to 2 days after delivery. The post mortem and histological features are supportive of a diagnosis of AFLP. Our patient presented 4 days after the onset of symptoms and even though she had delivered, the symptoms progressed unabated albeit due to lack of urgent comprehensive supportive care such as transfusion of blood products and dialysis. There is usually improvement of symptoms 1 to 2 days after delivery but for patients who are critically ill and in those in whom complications progress despite delivery, management should be carried out in the intensive care unit.

## Conclusion

AFLP is a rare, life-threatening complication of third trimester which requires a high index of suspicion for early diagnosis. Urgent delivery and maximum supportive care should be instituted to prevent poor outcomes.

## Data Availability

Not applicable.

## Abbreviations

AFLP: Acute fatty liver of pregnancy
ALP: Alkaline phosphatase
ALT: Alanine aminotransferase
ANC: Antenatal care
AST: Aspartate aminotransferase
EGA: Estimated gestational age
GGT: gamma glutamyl transpeptidase
HELLP: Haemolysis elevated liver enzymes low platelets
HIV: Human immunodeficiency virus
ICP: Intrahepatic cholestasis of pregnancy
PLT: Platelets
WBC: White blood cell count
